# A Comparative Study of Hesperetin, Hesperidin and Hesperidin Glucoside: Antioxidant, Anti-Inflammatory, and Antibacterial Activities In Vitro

**DOI:** 10.3390/antiox11081618

**Published:** 2022-08-20

**Authors:** Sung-Sook Choi, Sun-Hyung Lee, Kyung-Ae Lee

**Affiliations:** 1 R&D Center, Youngjin Bio Co., Suwon 16614, Korea or; 2 Department of Food and Nutrition, Duksung Women’s University, Seoul 01370, Korea; 3 Department of Food and Nutrition, Anyang University, Anyang 14028, Korea

**Keywords:** hesperetin, hesperidin, hesperidin glucoside, solubility, antioxidant, anti-inflammatory, antibacterial, in vitro

## Abstract

The antioxidant, anti-inflammatory and antibacterial activities of hesperetin, hesperidin and hesperidin glucoside with different solubility were compared in vitro. Hesperetin was prepared by enzymatic hydrolysis from hesperidin, and hesperidin glucoside composed of hesperidin mono-glucoside was prepared from hesperidin through enzymatic transglycosylation. Solubility of the compounds was different: the partition coefficient (log P) was 2.85 ± 0.02 for hesperetin, 2.01 ± 0.02 for hesperidin, and −3.04 ± 0.03 for hesperidin glucoside. Hesperetin showed a higher effect than hesperidin and hesperidin glucoside on radical scavenging activity in antioxidant assays, while hesperidin and hesperidin glucoside showed similar activity. Cytotoxicity was low in the order of hesperidin glucoside, hesperidin, and hesperetin in murine macrophage RAW264.7 cells. Treatment of the cells with each compound reduced the levels of inflammatory mediators, nitric oxide (NO), prostaglandin E2 (PGE2), tumor necrosis factor alpha (TNF-α) and interleukin-6 (IL-6). Hesperetin was most effective at relatively low concentrations, however, hesperidin glucoside was also effective at higher concentration. Hesperetin showed higher antibacterial activity than hesperidin in both Gram-positive and -negative bacteria, and hesperidin glucoside showed similarly higher activity with hesperetin depending on the bacterial strain. In conclusion, hesperetin in the form of aglycone showed more potent biological activity than hesperidin and hesperidin glucoside. However, hesperidin glucoside, the highly soluble form, has been shown to increase the activity compared to poorly soluble hesperidin.

## 1. Introduction

Hesperidin (3′,5,7-Trihydroxy 4′-methoxyflavanone 7-rutinoside, hesperetin 7-rutinoside) is found abundantly in citrus (family *Rutaceae*) fruits, such as lemons, limes, mandarins, oranges, and grapefruits. Structurally, it is a flavanone glycoside composed of the aglycone hesperetin and the disaccharide rutinose. Many studies have reported about the absorption, bioavailability, and pharmacokinetics of hesperidin. These studies suggest that hesperidin ingested orally in humans or animals is absorbed in the form of aglycone (hesperetin) after the removal of rutinose or via hesperetin 7-glucoside from hesperidin by bacterial enzymes in the intestine, followed by conversion to glucuronidated and sulfated metabolites that can be detected in blood or urine [1,2,3,4]. Both Hesperidin and hesperetin are well known as potent natural antioxidants effective in reducing oxidative stress. [5,6]. Hesperidin and hesperetin have been documented to exhibit a wide range of pharmacological properties, including anti-inflammatory, antimicrobial, anticarcinogenic, antithrombotic and anti-viral activity [7,8,9,10,11]. Recently, it has been demonstrated that hesperidin has a beneficial effect on the skin [12].

However, the low water solubility of hesperidin and its aglycone, hesperetin, limits its expansion to industrial applications such as food, cosmetic and pharmaceutical applications. Various studies have been conducted to improve the solubility and biological effects of flavonoids including hesperidin. Enhancement of the water solubility and antioxidant activity have been reported on hesperidin mixed with chitooligosaccharide [13]. It has also been reported that inclusion complexes of hesperidin or hesperetin with hydroxypropyl β-cyclodextrin increase solubility and antioxidant potential [14]. Glycosylation of hesperidin or hesperetin has been studied and is considered an efficient and promising method to increase solubility and bioavailability [15,16,17].

**Figure 1:**
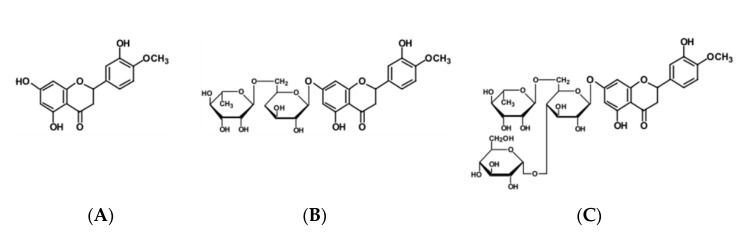
Structures of hesperidin and related compounds. (**A**) hesperetin, (**B**) hesperidin and (**C**) hesperidin glucoside (mono-glucoside).

The effects of natural or artificially additional sugar moiety of flavonoids on solubility changes have been extensively studied [18,19]; however, information about changes in the properties and efficacy is not fully understood. The biological effects of hesperetin, hesperidin and hesperidin glucoside varied depending on the concentration tested, solvent used, and test conditions such as in vitro or in vivo [20,21,22]. The purpose of this study is to investigate whether hesperetin, hesperidin and hesperidin glucoside with different solubility could have different effects on biological activity such as antioxidant, anti-inflammation, and antibacterial activity in vitro. The chemical structures of hesperidin and its aglycone, hesperetin, and its artificial glucoside, hesperidin mono-glucoside, are shown in Figure 1.

## 2. Materials and Methods

### 2.1. Materials and Chemicals

Refined hesperidin (>95%) separated from *Citrus aurantium* L. was obtained from IBT Co., Ltd. (Gunpo, Korea). Commercially available enzymes, cyclodextrin glucanotransferase (CGTase, Toruzyme^TM^) and glucoamylase (AMG^TM^) were products of Novozymes (Gladsaxe, Denmark), and a hydrolytic enzyme, Plantase AK^TM^, was obtained from Bision Biochem. Corp. (Seoul, Korea). Dextrin with dextrose equivalent 8–15 was purchased from Sigma-Aldrich Co. (St. Louis, MO, USA), and macroporous resin (Amberlite XAD-7) was purchased from Dow Chemical (Midland, MI, USA).

For antioxidant assays, 1,1-Diphenyl-2-picrylhydrazyl (DPPH) and 2,2′-azino-bis-(3-ethylbenzothiazoline-6-sulfonic acid) (ABTS) were purchased from Sigma-Aldrich Co. (St. Louis, MO, USA). For cell-based anti-inflammatory assays, 3-(4,5-Dimethylthiazol-2-yl)-2,5-diphenyltetrazolium bromide (MTT), lipopolysaccharide (LPS), and dimethyl sulfoxide (DMSO) were purchased from Sigma Chemical Co. (St. Louis, MO, USA). Dulbecco’s modified eagle’s medium (DMEM) and fetal bovine serum (FBS) were products of Hyclone (Logan, UT, USA). A detection kit for nitric oxide (NO) was purchased from iNtRON Biotechnology (Seongnam, Korea), and ELISA kit for prostaglandin E2 (PGE2) was purchased from Enzo Biochem. (New York, NY, USA), kits for tumor necrosis factor alpha (TNF-α) and interleukin-6 (IL-6) were purchased from Thermo Fisher Scientific. For antibacterial testing, resazurin was purchased from Sigma-Aldrich Co. (St. Louis, MO, USA), and culture media for bacterial growth were the products of BD Biosciences (Heidelberg, Germany).

### 2.2. Preparation of Hesperetin and Hesperidin Glucoside

Enzymatic hydrolysis and transglycosylation were performed based on the methods described in Baik et al. [23] and Choi et al. [24] with some modification. For the preparation of hesperetin, hesperidin powder was dissolved in 0.1 N NaOH solution at a concentration of 1% (*w*/*v*). Immediately after hesperidin was solubilized, the pH was adjusted to 7.0 and a hydrolytic enzyme was added to a final concentration of 1% (*w*/*v*). After reaction for 16 h at 50 °C and pH 6.5, the precipitate was harvested and solubilized in absolute ethanol. The ethanol solution was filtered through a filter (0.45 μm) to remove insoluble debris and the filtrate was evaporated and suspended again in water. The precipitate was harvested and dried.

For the preparation of hesperidin glucoside, hesperidin powder was dissolved in 0.1 N NaOH solution at a concentration of 1% (*w*/*v*). Immediately after hesperidin was solubilized, the pH was adjusted to 7.0, and dextrin was added in a ratio of 4:1 to hesperidin and CGTase was added at a final concentration of 5% (*w*/*v*). After reaction for 48 h at 50 °C and pH 6.5, glucoamylase was added at a concentration of 1% and reacted for 3 h at 40 °C and pH 4.5 to produce hesperidin mono-glucoside. The reactant was filtered using a filter (0.2 μm) and the filtrate was loaded on a column filled with an adsorption resin. After loading the sample, the column was washed with deionized water and eluted with 60% ethanol. The eluent was evaporated and concentrated at 50 °C, and five volumes of ethanol were added and cooled down to 5 °C or less for 24 h to induce precipitation. The precipitate was harvested and dried.

The conversion rate of hesperetin or hesperidin glucoside from hesperidin was calculated as the ratio of the amount of hesperidin reacted from the amount of hesperidin initially used.

$$\mathrm{Conversion}\;\mathrm{rate}\;\left(\%\right)=\frac{\mathrm{Amount}\;\mathrm{of}\;\mathrm{hesperidin}\;\mathrm{initially}\;\mathrm{used}\;-\;\mathrm{Amount}\;\mathrm{of}\;\mathrm{hesperidin}\;\mathrm{remained}\;\mathrm{after}\;\mathrm{reaction}}{\mathrm{Amount}\;\mathrm{of}\;\mathrm{hesperidin}\;\mathrm{initially}\;\mathrm{used}}\;\times \;100$$

### 2.3. Analysis of Hesperetin, Hesperidin and Hesperidin Glucoside

#### 2.3.1. Determination of Hesperetin, Hesperidin and Hesperidin Glucoside

The composition and content of hesperetin and hesperidin were analyzed using HPLC (Chromaster 5110, Hitachi, Tokyo, Japan). A C18 column (CAPCELL PAK C18 UG120 S5, 5 μm, 2.0 mm × 150 mm, OSAKA SODA, Osaka, Japan) was used and the mobile phases were water (0.1% formic acid) and acetonitrile (0.1% formic acid) binary eluents under gradient conditions: 0 min (77% water:23% acetonitrile), 3.5 min (40%:60%), 9 min (40%:60%), 23 min (40%:60%) and 24 min (77%:23%), 35 min (77%:3%). The flow rate was 0.3 mL/min, the column temperature was maintained at 25 °C, and the detection wavelength was 280 nm.

An amide column (TSKgel Amide-80, 5 μm, 4.6 × 250 mm, Tosoh Corp., Tokyo, Japan) was used for detection of hesperidin mono- to poly-glucosides in the process of transglycosylation of hesperidin. The mobile phases used were acetonitrile and water binary eluents under gradient conditions: 0 min (80% acetonitrile:20% water), 15 min (65%:35%), 30 min (60%:40%), 37 min (80%:20%) and 50 min (80%:20%). The flow rate was 0.8 mL/min, the column temperature was maintained at 25 °C, and the detection wavelength was 280 nm. In addition, the hesperidin content in the hesperidin glucoside samples were also measured according to the KFDA method [25]. Briefly, each sample was dissolved in deionized water to a concentration of 1% (*w*/*v*), then 1 mL aliquot was diluted to 100 mL with water and used as a sample solution. Hesperidin standard was solubilized in 0.5 N NaOH solution to a final concentration of 1% (*w*/*v*), then 1 mL aliquot was diluted to 100 mL with water and used as a standard solution. The sample and standard solution was analyzed via HPLC using CAPCELL PAK C18 column.

#### 2.3.2. Identification by FT-IR

Chemical properties of hesperetin, hesperidin and hesperidin glucoside were analyzed using a FT-IR Spectrometer (Nicolet iS5, Thermo Fisher Scientific, Waltham, MA, USA). Spectral scanning was taken in the wavelength region between 400 and 4000 cm^−1^ with a resolution of 2 cm^−1^.

#### 2.3.3. Identification by LC-MS

Hesperetin and hesperidin glucoside obtained through enzymatic reaction were identified by analyzing fragmentation in LC-MS-(MS) spectra. Chromatographic separation was conducted on an HPLC system (LC-20AD, Shimadzu, Kyoto, Japan) based on the HPLC analysis method described. LC/MS detection was conducted on Q-TOF Premierometer (Waters, Milford, MA, USA) system with an electrospray ion source in the positive mode. The ion transitions for confirmation and quantification were *m*/*z* 303 (precursor ion) → 153 (product ion) for hesperetin, and *m*/*z* 773 (precursor ion) → 303 (product ion) for hesperidin glucoside.

### 2.4. Determination of Partition Coefficient

The hydrophilic/hydrophobic properties of hesperetin, hesperidin and hesperidin glucosides were investigated measuring partition coefficients in an octanol–water system [26,27]. Samples (500 μM each) were dissolved in equal volumes of water and n-octanol, and the mixture solution was maintained in the dark at room temperature to reach partition equilibrium. After incubation for about 20 h, the sample concentrations were determined by HPLC. Octanol–water partition coefficients (log P) were calculated as log ratio of the sample concentration in the octanol phase to the concentration in the aqueous phase:

$$\mathrm{Partition}\;\mathrm{coefficient}\;\mathrm{Log}\;\left(P\right)=\frac{\mathrm{Log}\;\left[C\right]\mathrm{octanol}\;}{\mathrm{Log}\;\left[C\right]\mathrm{water}}$$

where [C]octanol is the concentration of the sample in the n-octanol phase and [C]water is the concentration of the sample in the aqueous phase.

### 2.5. Determination of Antioxidant Activity

#### 2.5.1. DPPH Radical Scavenging Assay

DPPH radical scavenging assay was performed based on the method of Blois [28] and Ratha et al. [29] with some modification. DPPH was solubilized in absolute ethanol to a concentration of 0.4 mM, and 0.2 mL aliquot of diluted sample was mixed with 3.8 mL of the DPPH solution. When the reactant color was stabilized, after reacting for 30 min, the absorbance was measured at 525 nm in a UV/Vis Spectrophotometer (S22, Biochrom, Cambridge, UK). The DPPH radical scavenging activity was expressed as a percentage of the difference between samples and controls.

#### 2.5.2. ABTS Radical Scavenging Assay

ABTS radical scavenging assay was performed based on the method of Re et al. [30] with some modification. ABTS radical (7 mM) in potassium persulfate solution (140 mM) was diluted with absolute ethanol until the absorbance at 734 nm reached 0.7 ± 0.02, and it was used as ABTS+ reagent. An aliquot of 0.2 mL sample was mixed with 5 mL of ABTS+ reagent and reacted for 6 min at room temperature. The absorbance of the reactant was measured at 734 nm in a UV/Vis Spectrophotometer (S22, Biochrom, UK). The ABTS radical scavenging activity was expressed as a percentage of the difference between samples and controls.

### 2.6. Determination of Effects on Inflammatory Mediators and Pro-Inflammatory Cytokines

#### 2.6.1. Cell Culture, Cytotoxicity

RAW 264.7 cells, a macrophage cell line, were provided from Korea Cell Line Bank (Seoul, Korea). Cells were cultured in DMEM with 10% FBS and penicillin/streptomycin (100 μg/mL each). The cytotoxicity of hesperetin, hesperidin and hesperidin glucoside was assessed by MTT assay [31]. In brief, cells were cultured in 96 wells at a density of approximately 5 × 10^4^ cells/well and then incubated for 24 h in a CO_2_ incubator. Different concentrations of hesperetin, hesperidin and hesperidin glucoside were added to the wells and maintained for 1 h, then LPS was added to a final concentration of 1 μg/mL and incubated for 24 h at 37 °C. After the removal of the medium in the wells, 100 μL of MTT solution was added to each well and maintained for 2 h at 37 °C. Then DMSO was added to the wells and the absorbance was measured at 540 nm in a microplate reader (Epoch2, Bio Tek, Winooski, VT, USA). Cell viability was expressed as a percentage of the difference in absorbance at 540 nm of sample compared to the control.

#### 2.6.2. Measurement of Inflammatory Mediators

Levels of NO and PGE2 were measured in RAW 264.7 cells with/without treatment of hesperetin, hesperidin and hesperidin glucoside. Cells were pre-cultured in 96 wells at a density of 5 × 10^4^ cells/well and treated with different concentrations of hesperetin, hesperidin and hesperidin glucoside for 1 h. LPS (final concentration of 1 μg/mL) was added to induce inflammation and incubated for 24 h at 37 °C. NO level in the media was measured using an NO detection kit according to the manufacturer’s instruction. The absorbance was measured at 540 nm in a microplate reader.

Cell culture and sample treatment conditions for PGE2 assay were almost the same as those used in NO assay. PGE2 levels in the media were measured using an ELISA kit according to the manufacturer’s experimental protocol. The absorbance was measured at 405 nm in a microplate reader.

#### 2.6.3. Measurement of Pro-Inflammatory Cytokine Levels

Levels of TNF-α and IL-6 were measured in RAW 264.7 cells with/without treatment of hesperetin, hesperidin and hesperidin glucoside. Cell culture and sample treatment conditions for TNF-α and IL-6 assay were almost the same as those used in NO assay. The concentrations of TNF-α and IL-6 in the media were measured, respectively, using commercial ELISA kits according to the manufacturer’s experimental protocols. The absorbance value was read at a wavelength of 450 nm in a microplate reader.

### 2.7. Determination of Antibacterial Activity

#### 2.7.1. Microorganisms

In vitro antibacterial activity was tested against Gram-positive *Staphylococcus aureus* (KCTC 3881), *Bacillus cereus* (ATCC 21772) and Gram-negative *Escherichia coli* (KCTC 2571), *Pseudomonas aeruginosa* (KCTC 2513). Tryptic soy agar was used for cultivation of *S. aureus* and *P. aeruginosa*, respectively, and nutrient agar was used for *E. coli* and *B. cereus*, respectively.

#### 2.7.2. Determination of Minimum Inhibitory Concentration (MIC)

The MIC value was determined based on the broth micro-dilution method [32,33,34] with some modifications. For the MIC test, each growth medium without agar was used for bacterial cultivation. Briefly, the culture broth of each bacterial strain was diluted with the same medium. Two-fold serial dilutions of hesperetin, hesperidin, and hesperidin glucosides were taken 10 µL in 96-well microplates containing 90 µL of the culture broth. After 24 h incubation, 1 mg/mL of resazurin solution (10 µL) was added to each well and the plate was then incubated for 2 h. The MIC value was determined as the lowest concentration of the sample that prevented a color change of resazurin (blue to pink) when it is reduced.

#### 2.7.3. Determination of Minimal Bactericidal Concentration (MBC)

After the MIC test, the MBC value was investigated by subculturing each well in which no visible growth occurred to an agar medium. After 24 h incubation at 37 °C, the bacterial colony on the agar plate was counted. The lowest concentration at which the sample eliminated all bacteria represented the MBC. The MBC is identified by determining the lowest concentration of the sample that reduces the viability of the initial bacterial inoculum by 99.9%.

### 2.8. Statistical Analysis

Most of the experimental procedures were performed in triplicate and repeated three times. Values were expressed as mean ± standard deviation. One-way analysis of variance (ANOVA) was performed using SPSS software (version 22, SPSS Inc., Chicago, IL, USA). Duncan’s multiple range test was used to test for significant differences between the treatments at *p* < 0.05.

## 3. Results

### 3.1. Preparation of Hesperetin and Hesperidin Glucoside

#### 3.1.1. Enzymatic Conversion of Hesperidin to Hesperetin

Hesperetin, the aglycone of hesperidin, was obtained by removal of the rhamnosyl glucose moiety. The reaction was conducted using a hydrolase containing various glucosidase activities. The conversion rate to hesperetin was high, showing 96.25 ± 2.21%. Hesperetin obtained through the enzymatic hydrolysis was separated and purified from unreacted hesperidin and sugars by repeated solubilization in absolute ethanol and filtration with a 0.2 μm membrane filter. After subsequent crystallization in water, highly purified hesperetin with more than 95% content was obtained.

#### 3.1.2. Enzymatic Conversion of Hesperidin to Hesperidin Glucoside

**Figure 2:**
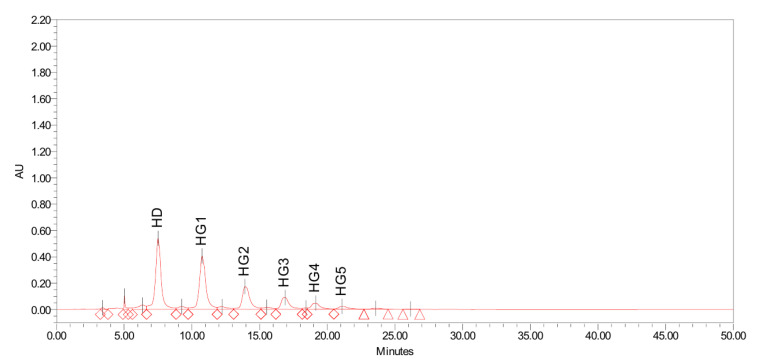
HPLC profiles of hesperidin glucosides synthesized during enzymatic transglycosylation. The x- and y-axis represents the retention time (min) and arbitrary unit (AU), respectively. Abbreviation, HD; hesperidin, HG1; hesperidin mono-glucoside, HG2; hesperidin di-glucoside, HG3; hesperidin tri-glucoside, HG4; hesperidin tetra-glucoside, HG5; hesperidin penta-glucoside.

The enzymatic synthesis of hesperidin glucosides was performed using dextrin as a glucose donor and hesperidin as an acceptor. As shown in Figure 2, various hesperidin glucosides were obtained through transglycosylation by CGTase. The glucose units bound to hesperidin were up to 5–6 glucose units after the reaction. The rate of enzymatic conversion from hesperidin to hesperidin glucoside in this study was 76.04 ± 4.97%. The obtained hesperidin poly-glucosides were then treated with a glucoamylase to remove the glucose units from hesperidin poly-glucosides, leaving hesperidin mono-glucoside. Hesperidin mono-glucoside obtained through a series of enzyme reactions was separated from residual sugars and unreacted hesperidin by centrifugation/filtration and purified using a column filled with adsorption resin. High purity hesperidin glucoside of 95% or more was obtained through column chromatographic separation and crystallization.

### 3.2. Identification of Hesperetin and Hesperidin Glucoside

#### 3.2.1. FT-IT Spectra of Hesperetin, Hesperidin and Hesperidin Glucoside

**Figure 3:**
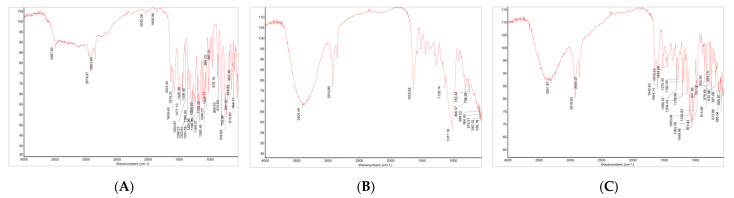
FT-IR spectra of hesperetin, hesperidin and hesperidin glucosides. (**A**) Hesperetin, (**B**) Hesperidin, (**C**) hesperidin glucoside (mono-glucoside).

In the FT-IR spectra of hesperetin (Figure 3), hesperidin and hesperidin glucoside, the characteristic absorption bands were present, and then identified, respectively, using OMNIC software library (Thermo Fisher Scientific, Waltham, MA, USA).

#### 3.2.2. Mass Spectral Fragmentation of Hesperetin, Hesperidin and Hesperidin Glucoside

The hesperetin obtained by the enzymatic hydrolysis was identified through LC-MS-MS analysis, and hesperidin glucoside obtained by the enzymatic transglycosylation was identified through LC-MS analysis.

**Figure 4:**
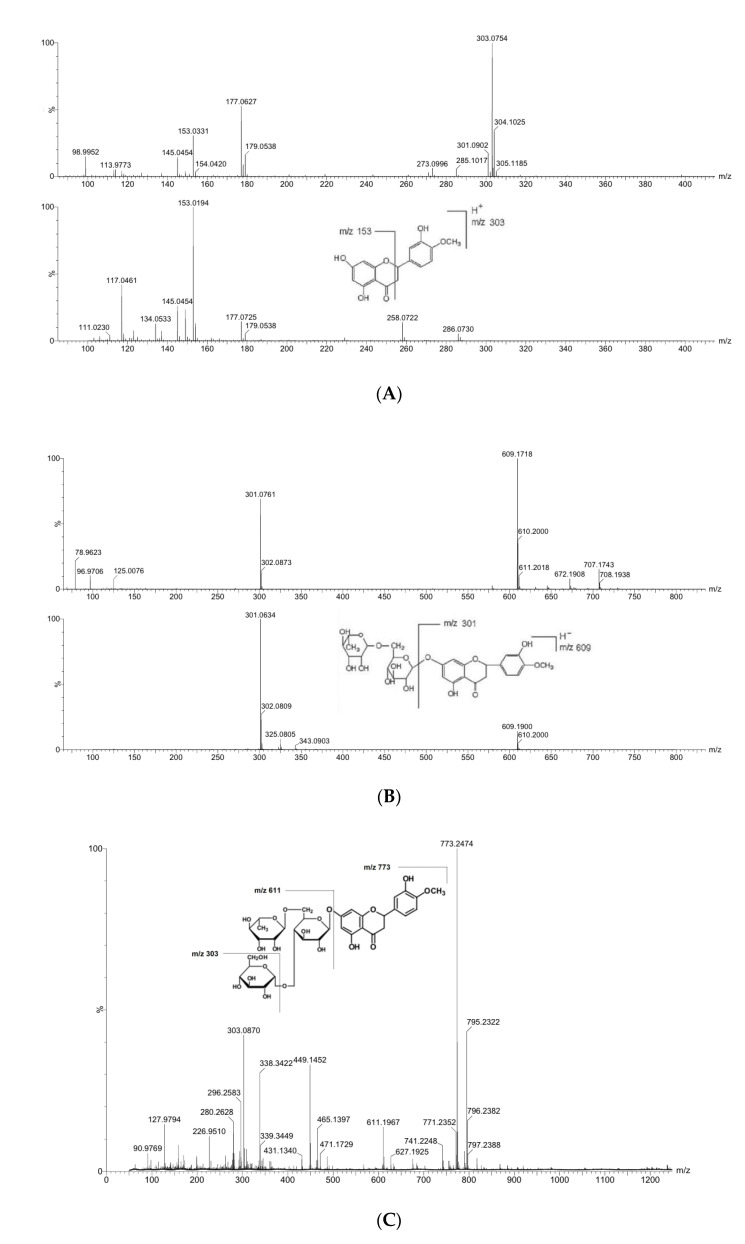
Mass spectra and fragmentation of prepared hesperetin, hesperidin and hesperidin glucoside by LC-MS-(MS) analysis. (**A**) Spectra of hesperetin, (**B**) spectra of hesperidin, (**C**) spectra of hesperidin glucoside.

It was shown in the spectra, the molecular ion of hesperetin (protonated) at *m*/*z* 303.0754 produced the fragment ion at *m*/*z* 153.0194 by breaking down (Figure 4A). The molecular ion of hesperidin at *m*/*z* 609.1900 produced the fragment ion at *m*/*z* 301.0634 by losing disaccharide rutinose (Figure 4B). The protonated molecular ion of hesperidin glucoside (mono-glucoside) at *m*/*z* 773.2474 produced the fragment ion at *m*/*z* 611.1967 by losing one glucose moiety, and also the fragment ion at *m*/*z* 303.0870 by losing a glucose and rutinose moiety (Figure 4C).

### 3.3. Partition Coefficient of Hesperetin, Hesperidin and Hesperidin Glucosides

Hesperetin, an aglycone form, is hardly soluble in water and hesperidin, and its glucoside with rutinose (rhamnosyl glucose), is still poorly soluble in water. The solubility of glucosylated hesperidin, whether hesperidin mono-glucoside or hesperidin poly-glucoside, increased drastically compared to hesperidin. The solubility of hesperetin, hesperidin and hesperidin glucoside (mono-glucoside) were determined by the octanol–water partition coefficient. The Log P value of hesperetin was a little higher than the value of hesperidin, but the value of hesperidin glucoside was much lower than those of hesperetin and hesperidin (Table 1). It can be estimated that hesperetin and hesperidin are very insoluble in water, but hesperidin glucoside has much higher water solubility compared to hesperetin and hesperidin.

### 3.4. Antioxidant Activity of Hesperetin, Hesperidin and Hesperidin Glucoside

**Figure 5:**
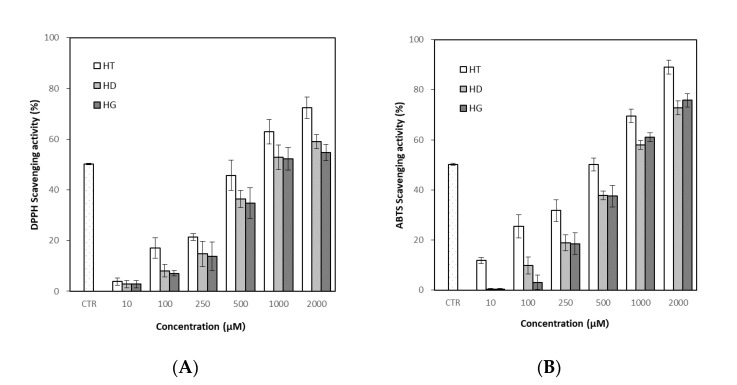
Antioxidant activity of hesperetin, hesperidin and hesperidin glucoside. Results were expressed as a mean ± SD (*n* = 3). (**A**) 1,1-diphenyl-2-picrylhydrazyl (DPPH) radical scavenging activity, (**B**) 2,2′-azino-bis-3-ethylbenzthiazoline-6-sulphonic acid (ABTS) radical scavenging activity. CTR+; positive control (ascorbic acid), HT; hesperetin, HD; hesperidin, HG; hesperidin glucoside.

Radical scavenging activity of DPPH and ABTS was measured to evaluate antioxidant activity. In DPPH assay, treatment of hesperetin, hesperidin and hesperidin glucoside showed DPPH radical scavenging activity in a concentration-dependent manner (Figure 5A). Hesperetin showed a higher effect than hesperidin and hesperidin glucoside, and hesperidin and hesperidin glucoside did not show a significant difference. The mean scavenging concentration (SC_50_, representing 50% of scavenging) was 525.18 ± 1.02 μM for hesperetin, 896.21 ± 0.15 μM for hesperidin, 911.00 ± 0.14 μM hesperidin glucoside, respectively, while the SC_50_ of ascorbic acid as a positive control was 61.78 ± 0.02 μM.

The results of the ABTS assay were similar to those of the DPPH assay, and the antioxidant effect of hesperetin showed a higher effect than hesperidin and hesperidin glucoside, but hesperidin and hesperidin glucoside did not show a significant difference (Figure 5B). The SC_50_ of ABTS assay was found to be 489.01 ± 0.09 μM for hesperetin, 796.02 ± 0.12 μM for hesperidin and 715.43 ± 0.14 μM for hesperidin glucoside, respectively, while the SC_50_ of ascorbic acid as a positive control was 70.63 ± 0.08 μM.

### 3.5. Effects of Hesperetin, Hesperidin and Hesperidin Glucoside on Cell Viability

**Figure 6:**
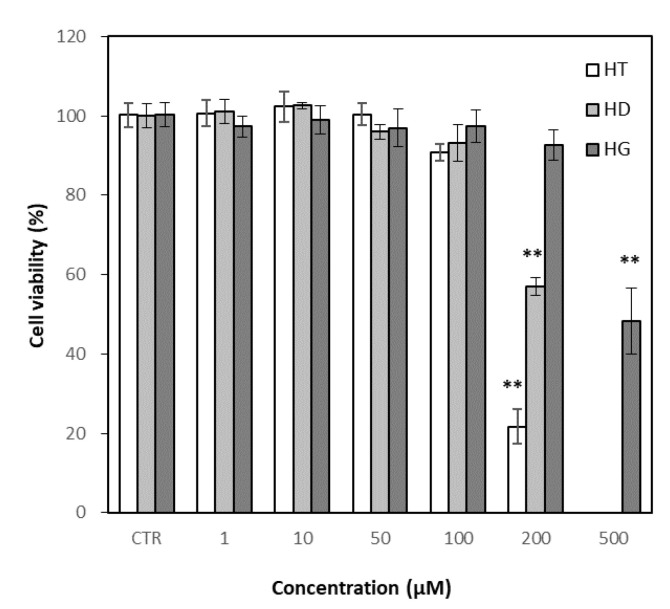
Effect of hesperetin, hesperidin and hesperidin glucoside on RAW 264.7 cell viability. Cells were treated with hesperetin, hesperidin and hesperidin glucoside, respectively, for 1 h and incubated for 24 h with LPS (final concentration of 1 μg/mL. Results were represented as mean ± SD (*n* = 3). Significant differences from the control (** *p* < 0.01). CTR; Control (with LPS), HT; hesperetin, HD; hesperidin, HG; hesperidin glucoside.

The cytotoxicity of hesperetin, hesperidin and hesperidin glucoside on RAW 264.7 cells was evaluated based on MTT assay. Hesperidin glucoside showed much less cytotoxicity than hesperidin in RAW 264.7 cells, while hesperetin showed more toxicity than hesperidin (Figure 6). Hesperidin glucoside did not induce significant cell death at the concentration at 200 μM, while hesperidin and hesperetin showed a marked cytotoxicity at the concentration. Anti-inflammatory assays were performed up to concentrations of 100 μM for hesperidin and hesperetin, and 200 μM for hesperidin glucoside.

### 3.6. Effects of Hesperetin, Hesperidin and Hesperidin Glucoside on Inflammation

#### 3.6.1. Effects on NO and PGE2 Levels

**Figure 7:**
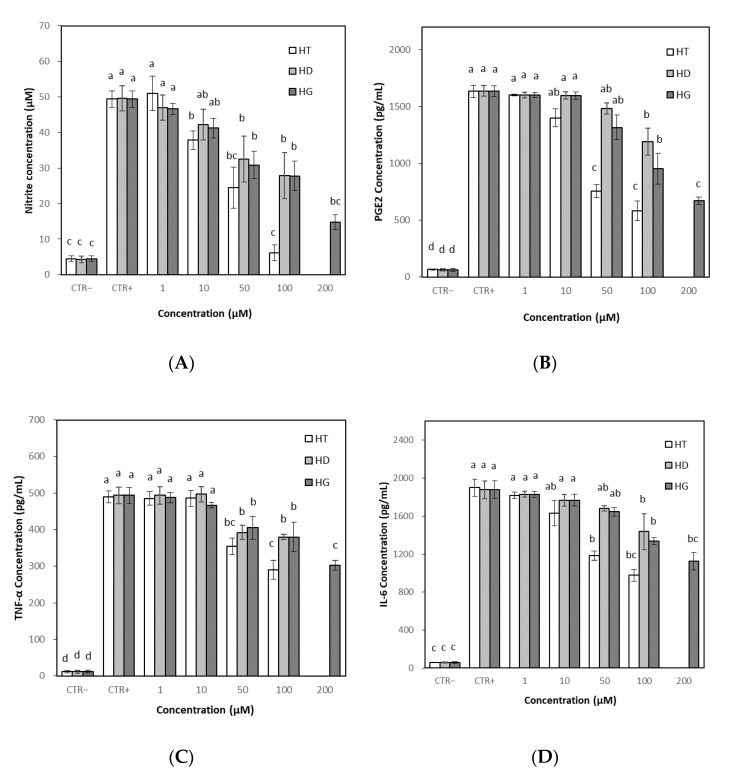
Effect of hesperetin, hesperidin and hesperidin glucoside on the production of nitric oxide (NO), Prostaglandin E2 (PGE2), tumor necrosis factor alpha (TNF-α) and interleukin 6 (IL-6). RAW 264.7 cells were cultured with different concentrations of hesperetin, hesperidin and hesperidin glucoside for 24 h with LPS (1 μg/mL). Results were represented as mean ± SD (*n* = 3). (**A**) Effect on NO levels; (**B**) Effect on PGE2 levels; (**C**) Effect on TNF-α levels; (**D**) Effect on IL-6 levels. Different letters (a, b, c, d) above the bars indicate significant differences (*p* < 0.05), where letters ab or bc indicate the intermediate significance between a and b, or b and c, respectively. CTR−; Negative control, CTR+; Positive control (with LPS), HT; hesperetin, HD; hesperidin, HG; hesperidin glucoside.

NO and PGE2 are well-known inflammatory mediators, and their overproduction can play an important role in the inflammatory process [35,36,37]. The effects of the hesperetin, hesperidin and hesperidin glucoside on NO levels were evaluated in RAW 264.7 cells. The stimulation of LPS to the cells induced significant NO production compared to the untreated control. Treatment with hesperetin, hesperidin or hesperidin glucoside began to suppress NO production from 10 μM, but the NO production was markedly decreased by treatment with hesperetin at concentrations up to 100 μM (Figure 7A). Hesperidin glucoside effectively reduced NO levels at a higher concentration of 200 μM. Treatment with hesperetin began to suppress PGE2 production from 10 μM. PGE2 production was also decreased by hesperidin and hesperidin glucoside at concentrations up to 100 μM. Hesperidin glucoside effectively reduced PGE2 levels at a high concentration of 200 μM, which was comparable to 50–100 μM of hesperetin. Similar to the effect on NO, hesperetin effectively reduced PGE2 levels compared to hesperidin and hesperidin glucoside (Figure 7B).

#### 3.6.2. Effects on TNF-α and IL-6 Levels

Pro-inflammatory cytokines, such as TNF-α and IL-6, were quantified in RAW 264.7 cells to evaluate the anti-inflammatory properties of hesperetin, hesperidin and hesperidin glucoside. The stimulation of LPS to the cells induced significant TNF-α and IL-6 production compared to the untreated control. Hesperetin, hesperidin and hesperidin glucoside suppressed TNF-α and IL-6 production, respectively, at over 50 μM. As in the NO and PGE2 production, hesperetin showed the highest reducing effect on TNF-α and IL-6 production. Treatment with hesperetin, hesperidin or hesperidin glucoside reduced the cytokine production to some extent, but drastic effects were not observed at tested concentrations up to 100 μg/mL except for hesperetin (Figure 7C,D).

### 3.7. Antibacterial Activity of Hesperetin, Hesperidin and Hesperidin Glucoside

#### 3.7.1. Effects on MIC

Hesperetin, hesperidin and hesperidin glucoside showed a higher antibacterial effect on Gram-positive bacteria than on Gram-negative bacteria. Among the compounds tested, hesperetin effectively inhibited the bacterial growth showing MIC values of 125 μg/mL against *S. aureus*, 250 μg/mL against *B. cereus*, 500 μg/mL against *E. coli* and *P. aeruginosa* (Table 2). Hesperidin glucoside showed relatively higher antibacterial activity than hesperidin. However, considering that the molecular weight of hesperetin is about 303 Dalton and that of hesperidin glucoside is about 773 Dalton, the antibacterial activity of hesperidin glucoside is comparable to that of hesperetin, and much higher than that of hesperidin. In the negative control, sterile water had no inhibitory effect on any of the bacteria tested, but DMSO over 2% concentration had an effect on the growth of the bacteria. DMSO concentration was adjusted to a maximum 2% (*v*/*v*) for the experimental procedures, if necessary.

#### 3.7.2. Effects on MBC

As in the MIC results, the MBC values showed that hesperetin, hesperidin and hesperidin glucoside had higher bactericidal activity against Gram-positive bacteria than Gram-negative bacteria. The lowest MBC values were obtained from hesperetin against *S. aureus* and *B. cereus.* However, considering that the molecular weights of hesperetin and hesperidin glucoside are different more than twice, as in the case of MIC, the bacteriocidal activity of hesperidin glucoside is comparable to that of hesperetin, and much higher than that of hesperidin. (Table 3).

**Figure 8:**
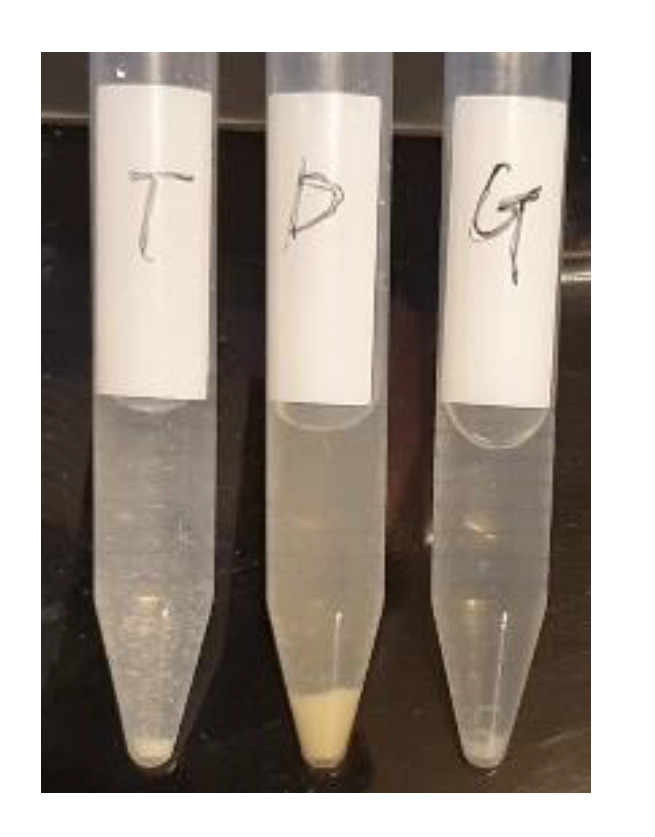
An example of precipitates containing microbial cells and insolubilized compounds in culture broth.

There were some precipitates in the bacterial culture broth at high concentration of hesperidin or hesperetin, because 2% of DMSO did not sufficiently solubilize hesperidin or hesperetin. On the other hand, hesperidin glucoside was completely solubilized in the culture broth (Figure 8).

## 4. Discussion

Hesperidin and hesperetin have been found as major flavonoids in many plants, especially in citrus fruits [38,39]. Citrus fruits contain a very low amount of hesperetin (hesperidin aglycone), and most of the flavanones exist as glucosides [40]. Hesperidin can be converted to hesperetin by removal of the sugar moiety when an infectious microorganism attacks the host plant or when ingested by an animal [41,42]. Although the aglycone form is generally considered the active form, there is controversy over whether natural flavonoid glycosides can be absorbed in the gastrointestinal tract, or whether they are hydrolyzed in the small intestine prior to absorption. Many studies have suggested that flavonoid aglycones permeate into the intestinal submucosal layer through passive diffusion [4,43]. The passive and pH-dependent transport of hesperetin was observed in isolated rabbit corneas [44]. It was also reported that hesperetin is absorbed by transcellular transport, which occurs mainly via proton-coupled active transport and passive diffusion [45]. Compared to hesperetin which is efficiently absorbed in the intestine, hesperidin is poorly transported via the paracellular pathway, and its absorption is dependent on conversion to hesperetin by intestinal microbial enzymes. The absorption and metabolism of flavonoids are complex processes that affects its bioavailability [46,47]. It has also been studied that orally ingested flavanones are absorbed as phase II metabolites, such as glucuronides and sulfates, in the small intestine, but many ingested flavanones reach and are absorbed in the large intestine as phase II metabolites or phenolic catabolites through the action of the intestinal microbiota [48,49,50]. Most reports have focused on hesperetin and its metabolites, but the absorption of hesperidin in its intact form or its metabolites was also observed in across Caco-2 cell monolayers, and in vivo [45,51]. Studies have shown that hesperidin glucoside or rutin glucoside can be absorbed after hydrolysis of the glycosylated sugar (glucose) and the original sugar moiety (rutinose), but absorption of rutin glucoside in its intact form has also been observed in rats [52,53].

Hesperidin is one of the commercially available and important flavonoids, but the application of hesperidin has been limited in many fields due to its extremely low water solubility. It has been reported that the solubility change of flavonoids could affect their biological efficacies [54,55]. Eriocitrin, a flavanone similar to hesperidin with two hydroxyl groups has shown a better solubility and efficacy compared to hesperidin [4,56]. It was reported that the intake of eriocitrin rich-lemon extract provides more circulating phase-II flavanone metabolites and higher concentrations than after consuming hesperidin-rich extracts [4]. Glycosylated flavonoids have been developed and studied for their improved solubility and efficacy [57]. On the contrary, it was also reported that the biological activities increase when flavonoid glucoside, a storage form in plants, is converted to aglycone, an activated form [58,59]. However, there are few reports comparing the various effects of hesperetin, hesperidin and hesperidin glucoside simultaneously [12,53,60]. The study could be important for the application of the compounds in non-digestive systems such as topical applications or subcutaneous injections, etc. In this study, we investigated the antioxidant, anti-inflammatory, and antibacterial effects in vitro to assess the differences between the compounds with different solubility.

Although flavonoids are known as antioxidants, it has been reported that flavonoids can act not only as antioxidants but also as pro-oxidants, depending on their concentration and environment [61,62]. Antioxidant assays, which are related to anti-inflammatory and many other biological activities [63,64], were performed prior to the determination of anti-inflammatory activity. The structure and activity relationships of flavonoids as antioxidants were extensively studied [65]; the antioxidant activity of flavonoids depends on the number and location of the hydroxyl moieties, the presence of double bonds in ring C, 3- and 5-hydroxy groups and the glycosylation type and position, etc. It was reported that 3′ OH group in flavonoid structure, including hesperidin, is important for antioxidant activity [66]. In addition to the aglycone structure, the antioxidant activity of flavonoids was also variable depending on glycoside structure. Increased solubility of hesperetin-7-glucoside compared with hesperidin results in more efficient prevention of bone loss in rats [67]. In this study, hesperetin, the aglycone form, showed higher activity than hesperidin and hesperidin glucoside in both the DPPH and ABTS radical scavenging assay. However, hesperidin and glucosyl hesperidin did not show a significant difference despite different solubility. In the DPPH and ABTS radical scavenging assays, the structure giving the difference in solubility does not seem to have a significant effect, and the basic structure of the flavonoid seems to have more influence. However, changes in solubility or toxicity that maintain biological activity may increase the applicability of hesperidin. Sahiner et al. [68] have suggested the potential of poly naringin particles with lower cytotoxicity with antioxidant activity as an oral supplement.

Inflammation is an important biological response to maintain body homeostasis. However, excessive inflammation can cause tissue damage, resulting in destructive effects on the body [69]. Inflammation and oxidative stress are closely related to various life-threatening diseases including cancer, neurodegenerative and cardiovascular diseases [70,71]. Oxidative stress can activate some transcription factors, and the transcription factors can induce the expression of many genes including those for pro-inflammatory cytokines [72]. Flavonoids are known as natural anti-inflammatory agents, and various studies are being conducted to understand the properties and efficacies of flavonoids, including hesperidin [73]. Giménez-Bastida et al. [74] studied that hesperetin and its phase II metabolites inhibited human aortic endothelial cell migration in the presence of TNF-α, and decreased plasminogen activator inhibitor-1-level in the cells. Ávila-Gálvez et al. [75] reported that aglycones of polyphenols including hesperetin exerted dose-dependent antiproliferative and estrogenic/antiestrogenic activities in breast cancer cells but both their glucuronide and sulfate conjugates lacked these activities. In this study, using RAW 264.7 cells, hesperetin, hesperidin and hesperidin glucoside showed concentration-dependent reductions in the levels of NO, PGE2, TNF-α and IL-6. Hesperetin had higher cytotoxicity than hesperidin and hesperidin glucoside, but also had a higher anti-inflammatory effect. Hesperidin and hesperidin glucoside did not show significant differences in their anti-inflammatory activities. The comparison could not be completed up to 200 μM because of the different cell viability of the three compounds, but hesperidin glucoside with relatively low cytotoxicity showed comparable effects at 200 μM to hesperetin. The result would be beneficial for the application of hesperidin glucosides.

Recent studies indicated that hesperidin and hesperetin possess antimicrobial activity [76]. The protective effects of hesperidin and hesperetin against toxicities induced by microbes and certain chemotherapy drugs have been widely investigated [77,78]. The exact mechanisms of their antimicrobial properties are not fully understood, but several mechanisms such as bacterial membrane disruption, inactivation of microbial enzymes, and activation of the host immune system, have been proposed [79]. However, many studies have suggested that increased lipophilicity may increase antimicrobial activity, that is, the aglycone form of flavonoids may be more effective than the glycoside form in inhibiting microbial growth [79,80]. In this study, hesperetin with high Log P value (poorly water soluble) showed high antibacterial activity against the Gram-positive and Gram-negative bacteria tested, but hesperidin glucoside, with very low Log P value (highly water soluble), also showed higher antibacterial activities than hesperidin with high Log P value. Lipophilicity of flavonoids is an important factor for antimicrobial activity, but high lipophilicity does not always lead to high antimicrobial activity in many cases [81,82,83]. In this study, 2% of DMSO did not sufficiently increase the solubility of hesperidin or hesperetin, and there were some precipitates in the bacterial culture broth at high concentration of hesperidin or hesperetin. On the other hand, hesperidin glucoside was completely solubilized in the culture broth. It has been suggested that hesperidin glucoside is absorbed more rapidly than hesperidin in rat intestines due to its high solubility [52]. The higher antimicrobial activity of hesperidin glucoside can be attributed to its higher solubility than hesperidin. It is under investigation whether hesperidin glucoside is converted to aglycone or other metabolites during the cultivation.

## 5. Conclusions

Hesperidin and hesperidin-derived compounds, hesperetin and hesperidin glucoside, were prepared and their solubility and biological efficacy were compared. Hesperidin glucoside showed a much higher water solubility than hesperidin and hesperetin. Hesperetin, the aglycone form, showed higher activity than hesperidin and hesperidin glucoside in both the DPPH and ABTS radical scavenging assay. Hesperetin also showed a higher anti-inflammatory effect in RAW 264.7 cells compared to hesperidin and hesperidin glucoside. In the antibacterial assay, hesperetin and hesperidin glucoside showed a similarly higher efficacy than hesperidin. It was estimated that the aglycone form of hesperidin, hesperetin, may have high biological activity, and that the increased solubility of hesperidin glucoside may affect biological activities such as antibacterial activity.

## Figures and Tables

**Table 1:** n-Octanol/water partition coefficients of hesperetin, hesperidin and hesperidin glucosides.

Compound	Log P_o/w_
Hesperetin	2.85 ± 0.05 ^a^
Hesperidin Hesperidin mono-glucoside	2.01 ± 0.02 ^b^ −3.04 ± 0.09 ^c^

Results were represented as a mean ± SD (*n* = 3). Different letters (^a^, ^b^ and ^c^) in the column are significant differences according to Duncan’s multiple range test (*p* < 0.05).

**Table 2:** Minimal inhibitory concentration (MIC) of hesperetin, hesperidin and hesperidin glucoside.

Microorganism	MIC Value (μg/mL)		
	Hesperetin	Hesperidin	Hesperidin Glucoside
*S. aureus* (KCTC 3881)	125	1000	500
*B.cereus* (ATCC 21772)	250	2000	500
*E. coli* (KCTC 2571)	250	>2000	1000
*P. aeruginosa* (KCTC 2513).	500	2000	1000

**Table 3:** Minimal bacteriocidal concentration (MBC) of hesperetin, hesperidin and hesperidin glucoside.

Microorganism	MBC Value (μg/mL)		
	Hesperetin	Hesperidin	Hesperidin Glucoside
*S. aureus* (KCTC 3881)	500	>2000	1000
*B.cereus* (ATCC 21772)	500	>2000	1000
*E. coli* (KCTC 2571)	1000	>2000	2000
*P. aeruginosa* (KCTC 2513).	2000	>2000	2000

## Data Availability

Data is contained within the article.
